# *In silico* SNP analysis of the breast cancer antigen NY-BR-1

**DOI:** 10.1186/s12885-016-2924-7

**Published:** 2016-11-18

**Authors:** Zeynep Kosaloglu, Julia Bitzer, Niels Halama, Zhiqin Huang, Marc Zapatka, Andreas Schneeweiss, Dirk Jäger, Inka Zörnig

**Affiliations:** 1 Clinical Cooperation Unit “Applied Tumor Immunity”, National Center for Tumor Diseases (NCT) and German Cancer Research Center (DKFZ), Heidelberg, Germany; 2 Department of Medical Oncology, National Center for Tumor Diseases (NCT) and University Hospital Heidelberg, Heidelberg, Germany; 3 Division of Molecular Genetics, German Cancer Research Center (DKFZ), Heidelberg, Germany; 4 Department of Obstetrics and Gynecology, National Center for Tumor Diseases (NCT) and University Hospital Heidelberg, Heidelberg, Germany; 5 Im Neuenheimer Feld 460, 69120 Heidelberg, Germany; 6 Im Neuenheimer Feld 580, 69120 Heidelberg, Germany

**Keywords:** NY-BR-1, Breast cancer, Antigen, SNPs, In silico

## Abstract

**Background:**

Breast cancer is one of the most common malignancies with increasing incidences every year and a leading cause of death among women. Although early stage breast cancer can be effectively treated, there are limited numbers of treatment options available for patients with advanced and metastatic disease. The novel breast cancer associated antigen NY-BR-1 was identified by SEREX analysis and is expressed in the majority (>70%) of breast tumors as well as metastases, in normal breast tissue, in testis and occasionally in prostate tissue. The biological function and regulation of NY-BR-1 is up to date unknown.

**Methods:**

We performed an *in silico* analysis on the genetic variations of the NY-BR-1 gene using data available in public SNP databases and the tools SIFT, Polyphen and Provean to find possible functional SNPs. Additionally, we considered the allele frequency of the found damaging SNPs and also analyzed data from an in-house sequencing project of 55 breast cancer samples for recurring SNPs, recorded in dbSNP.

**Results:**

Over 2800 SNPs are recorded in the dbSNP and NHLBI ESP databases for the NY-BR-1 gene. Of these, 65 (2.07%) are synonymous SNPs, 191 (6.09%) are non-synoymous SNPs, and 2430 (77.48%) are noncoding intronic SNPs. As a result, 69 non-synoymous SNPs were predicted to be damaging by at least two, and 16 SNPs were predicted as damaging by all three of the used tools. The SNPs rs200639888, rs367841401 and rs377750885 were categorized as highly damaging by all three tools. Eight damaging SNPs are located in the ankyrin repeat domain (ANK), a domain known for its frequent involvement in protein-protein interactions. No distinctive features could be observed in the allele frequency of the analyzed SNPs.

**Conclusion:**

Considering these results we expect to gain more insights into the variations of the NY-BR-1 gene and their possible impact on giving rise to splice variants and therefore influence the function of NY-BR-1 in healthy tissue as well as in breast cancer.

## Background

Breast cancer is one of the most common malignancies and a leading cause of death among women. Although early stage breast cancer can be effectively treated, there are limited numbers of treatment options available for patients with advanced and metastatic disease. Therefore new targets and strategies need to be developed. A novel breast cancer differentiation antigen, designated as New York-Breast-1 (NY-BR-1), was identified by a serological cloning strategy (SEREX) [1, 2] and could be a possible target for immunotherapy for breast cancer patients [3]. NY-BR-1, also known as ANKRD30A, is located on chromosome 10p11-p12. There are several transcripts existing, which contain between 36 and 42 exons. Although computational analyses have identified NY-BR-1 as being a potential transcription factor, the functional aspects of this 158.9 kDa protein are still unknown. NY-BR-1 protein was shown to be expressed in normal breast epithelia cells and in a majority of primary breast cancers [4, 5], while NY-BR-1 mRNA was detected predominantly in breast cancers [6, 7]. NY-BR-1 is over-expressed in over 70% of primary breast tumors and metastases [1] and additional details on the involvement of NY-BR-1 in breast cancer will lead to a better understanding of the underlying processes.

Genetic variation can have a major impact on gene function and the functional range of a gene cannot be fully understood without awareness of the potential variability within a gene [8]. To further understand the biological function and regulation of NY-BR-1 and its potential for therapeutic approaches, we performed an *in silico* analysis on the genetic variations of the NY-BR-1 gene.

Human genetic variants may occur in diverse nucleotide compositions, including single nucleotide polymorphisms (SNPs) and structural variants such as small insertions and deletions (indels) or large copy number variations. Among these, SNPs are the most prevalent form of human variation and it has been estimated that one SNP exists every 290 base-pairs in the human genome [9]. Evidences show that through SNPs a wide range of human diseases such as cancer or autoimmunity can be triggered [10, 11]. SNPs also might affect the pharmacokinetics and pharmacodynamics of certain drugs in cancer therapy [12]. The transcriptional regulation of a protein, its structure and its function can be affected by a single base substitution, deletion or insertion. Two groups of SNPs are known: synonymous (sSNP) and non- synonymous SNPs (nsSNP). The latter results in changes of the translated amino acid sequence.

A number of studies have shown associations between one or few SNPs and complex diseases, but until today it is not entirely clear how much impact SNPs have on certain traits in different populations.With the steadily increasing number of known human nsSNPs, there is also growing interest in identification of the subset that may affect protein function. Various types of features can be used to predict the functional impact of nsSNPs: physical and chemical properties of the affected amino acids, structural properties of the encoded protein, and evolutionary properties, which can be inferred from sequence alignments of homologous proteins [13]. SIFT (Sorting Intolerant from Tolerant) [14], PROVEAN (Protein Variation Effect Analyzer) [15] and PolyPhen-2 (Polymorphism Phenotyping v2) [16] are computational prediction methods which take several of these properties into account and calculate a score to predict whether a given nsSNP has a functional impact. We obtained all SNPs for the NY-BR-1 gene and investigated the nsSNPs for their functional impact by using these three prediction tools. We identified a small number of nsSNPs which seem to affect the protein function of NY-BR-1. Additionally, we used in house sequencing data to analyze whether certain SNPs are enriched in breast cancer patients.

## Methods

### SNP Mining

dbSNP is hosted by the National Center for Biotechnology Information (NCBI) and is the largest repository of SNP data with over 140 million submitted variations [17].

Another source of variation data is provided by the “The National Heart, Lung and Blood Institute” (NHLBI). With the aim of discovering novel genes and mechanisms contributing to heart, lung and blood disorders, the NHLBI started the Exome Sequencing Project (ESP) and a large and well-phenotyped population with over 200,000 individuals was assembled. The protein coding regions of each individual genome (i.e. exome) is sequenced and the variation data is made publicly available [18].

The Ensembl Variation database incorporates variation data from several sources including dbSNP and NHLBI ESP. We used the web interface MartWizard (http://www.biomart.org/) of the BioMart Central Portal which offers access and crosslinks a wide array of biological databases.

The Ensembl transcript ID ENST00000611781 of the ANKRD30A gene was used to retrieve all available germline variations together with the corresponding genomic coordinates, the variant descriptions, the validation status, and allele frequency. Using the variant descriptions, we filtered coding non-synonymous SNPs (nsSNPs), coding synonymous SNPs (sSNPs) and intronic SNPs.

Additionally, exome-sequencing data were provided of 55 breast cancer patients from an in-house sequencing project (Division of Molecular Genetics, German Cancer Research Center (DKFZ), Heidelberg, Germany, and Heidelberg Center for Personalized Oncology (HIPO)). We also analyzed this dataset and looked for SNPs which are recorded in dbSNP.

### Prediction of the functional impact of coding nsSNPs using SIFT

The prediction tool SIFT evaluates the functional impact of SNPs based on sequence homology. The prediction is based on the degree of conservation of each amino acid residue of the query sequence. To assess the degree of conservation, SIFT compiles a dataset of functionally related protein sequences by searching the protein databases UniProt and TrEMBL using the PSI-BLAST algorithm and builds an alignment of the found sequences and the query sequence. In the second step a normalized probability for each substitution at each position of the alignment is calculated and is then recorded in a scaled probability matrix. This scaled probability is also called the SIFT score and a substitution is considered to be tolerated if the score is greater than 0.05; those less than 0.05 are predicted to be deleterious. The SIFT approach assumes that a highly conserved position is intolerant to most substitutions, whereas a poorly conserved position can tolerate most substitutions.

### Prediction of the functional impact of coding nsSNPs using PROVEAN

The tool PROVEAN also uses an alignment approach to assesses the functional impact of SNPs. PROVEAN consists of two main steps. In the first step, a set of homologous and distantly related sequences from the NCBI NR protein database is collected using BLASTP. To remove redundancy, the collected sequences are clustered, based on a sequence identity of 80%. A so called supporting set of sequences is assembled by adding sequences from clusters most similar to the query sequence, until a sufficient number of clusters is reached in the supporting set. In the second step, for each sequence in the supporting sequence set, a delta score is computed using the BLOSUM62 substitution matrix. For each cluster, an average delta score is computed, and the averaged delta scores are again averaged among all clusters. This unbiased averaged delta score is the final PROVEAN score.

The PROVEAN approach assumes that a variation, which reduces similarity of protein A to the homologous or distantly related protein B, is more likely to cause a damaging effect. Thus, the impact of a variation on protein function can be measured as the change in alignment score, the delta score. Low delta scores are interpreted as variations leading to a deleterious effect on protein function, while high delta scores are interpreted as variations with neutral effect.

The tools SIFT and PROVEAN are available online at http://sift.jcvi.org/ and http://provean.jcvi.org/, respectively. On the website, we used the tool PROVEAN Human Genome Variants, which provides PROVEAN and SIFT predictions for a list of human genome variants. We submitted the list of genomic coordinates and variants of our filtered 191 nsSNPs, and chose the default threshold of delta score < = −2.5 to detect deleterious variations.

### Prediction of the functional impact of coding nsSNPs using PolyPhen-2

PolyPhen-2 combines information on sequence features, multiple alignments with homologous proteins, and structural parameters to predict the impact of a SNP on protein function.

For sequence-based assessment, PolyPhen-2 tries to identify the query as an entry in the UniProtKB/Swiss-Prot database. Using the feature table of the corresponding entry, PolyPhen-2 checks if a given SNP occurs at functional relevant site, e.g. if the SNP lies within a transmembrane, signal peptide, or binding region.

Similar to SIFT, PolyPhen-2 also assesses the degree of conversation of the position where the SNP occurs by utilizing a multiple sequence alignment of homologous sequences. For each variant PolyPhen-2 calculates a position-specific independent counts (PSIC) score. The PSIC score difference between the two variants describes the impact of a particular amino acid substitution: the higher the PSIC score difference, the higher functional impact the substitution is likely to have.

A BLAST query of the query sequence against protein structure databases is carried out to identify corresponding 3D protein structures. If corresponding structures are found, they are used to assess, whether the SNP is likely to destroy the hydrophobic core, interactions with ligands or other important features of the protein.

Finally, all parameters are taken together and empirical prediction rules are applied to make the final decision, whether the SNP is damaging or benign.

PolyPhen-2 is available online at http://genetics.bwh.harvard.edu/pph2/. We used the option ‘Batch query’ and submitted the list of genomic coordinates and variants of our filtered 191 nsSNPs.

### DNA Sequencing and Analysis

The exon-sequencing library was prepared according to Agilent SureSelect Human All Exon V5 + UTRs protocol. Paired-end sequencing (2*101 bp) was carried out with Illumina Hiseq-2000 instruments. Paired-end sequencing reads were mapped to human genome reference assembly (hg19) with Burrows-Wheeler Aligner (BWA-v0.6.2) [19]. SAMtools mpileup (version-0.1.19) and bcftools (version-0.1.19) [20] were used to detect SNVs. Additional filtering step to remove possible artefacts was previously described [21]. Alignments on the NY-BR-1 gene only were extracted for this study and SNP states called at respective positions.

## Results

### SNP Mining

In the Ensembl BioMart database 2898 SNPs are recorded for the ANKRD30A transcript ENST00000611781. 2880 of these were imported from dbSNP and 18 from NHLBI ESP. 1832 SNPs have been validated by independent submissions or frequency/genotype data. However, the clinical significance has not been determined yet for any of the SNPs.

**Fig. 1:**
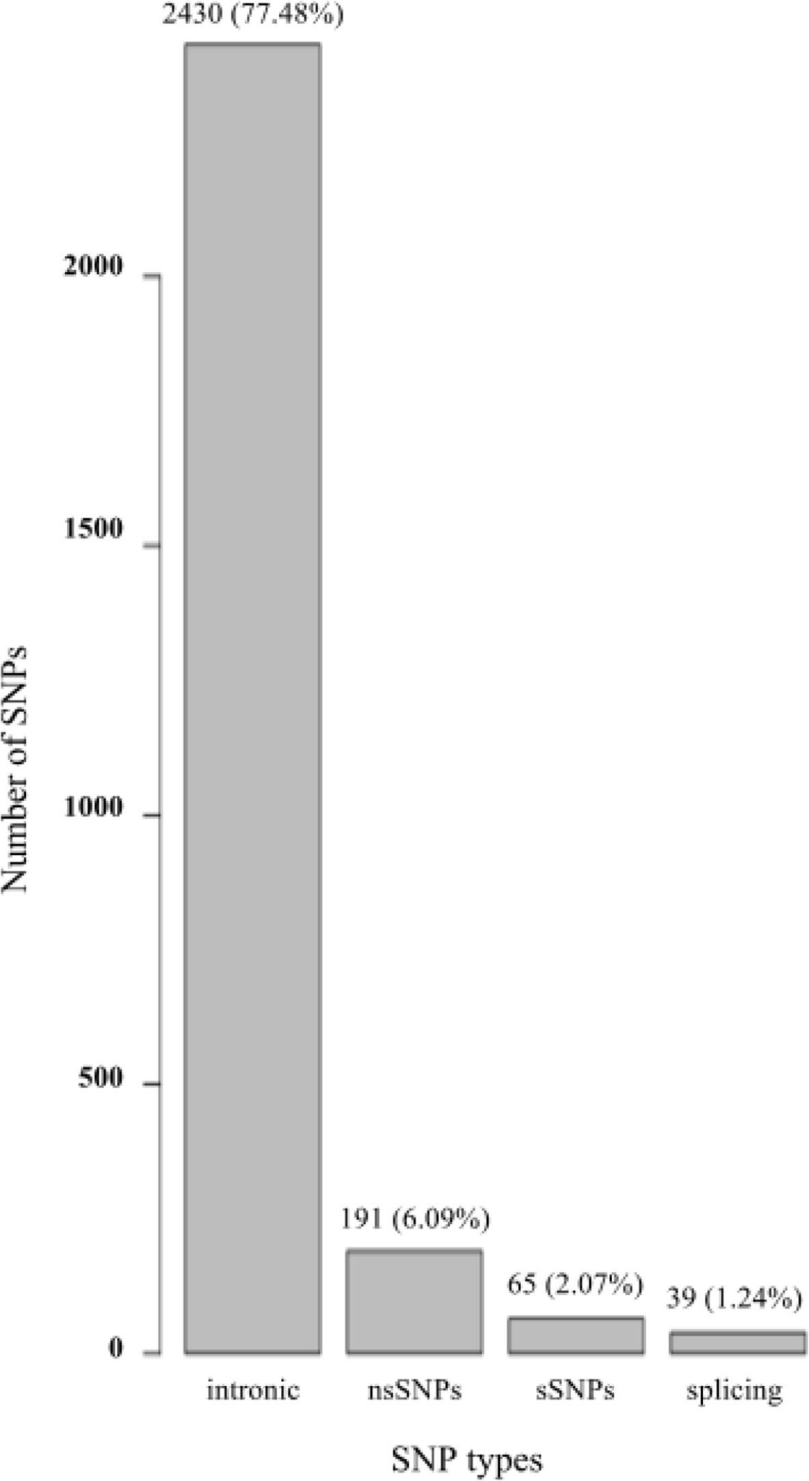
Graphical representation of distribution of intronic SNPs, non-synoymous SNPs (nsSNPs), synonymous SNPs (sSNPs), and SNPs at splicing sites for the NY-BR-1 gene, based on the dbSNP and NHLBI ESP databases

Out of all 2898 SNPs, 65 (2.07%) were sSNPs, 191 (6.09%) were nsSNPs, and 2430 (77.48%) occurred in intronic regions (Fig. 1). 40 of the downloaded SNPs are annotated as splice region variants in dbSNP. We selected nsSNPs for our investigation.

### Deleterious nsSNPs predicted by SIFT

Among the 191 analyzed nsSNPs, 79 nsSNPs were identified to be damaging with a tolerance index score > = 0.5. Ten nsSNPS showed a highly damaging tolerance index score of 0.00, namely rs200639888, rs372199195, rs144539033, rs369532435, rs199571878, rs376821949, rs267602482, rs201234943, rs367841401, and rs377750885. Nine nsSNPs had a tolerance index score of 0.001, nine nsSNPs had a score of 0.002, and five had a score of 0.003. The remaining nsSNPs contained tolerance index scores varying between 0.004 and 0.048.

### Damaging nsSNPs predicted by PROVEAN

28 nsSNPs out of the analyzed 191 nsSNPs were predicted to be deleterious with a delta score of < = −2.5. 10 nsSNPs showed a highly deleterious score of < − 4.00: rs200639888 (−5.962), rs61737412(−5.030), rs201943652(−4.758), rs189195791(−6.263), rs367841401 (−4.465), rs185294248(−4.366), rs374753521 (−4.184), rs371981371 (−4.603), rs377750885(−5.87), and rs201764363(−4.025).

20 nsSNPs were predicted as damaging variations by SIFT and PROVEAN. rs200639888, rs367841401, and rs377750885 were predicted to be highly damaging by SIFT with a tolerance index score of 0.00 and are also predicted to be highly deleterious by PROVEAN with delta scores of −5.962 and −4.465, and −5.87 respectively.

### Damaging nsSNPs predicted by PolyPhen

Out of the 171 nsSNPs submitted to the PolyPhen-2 server, 102 nsSNPs were considered to be damaging: 44 nsSNPs were predicted to be ‘probably damaging’ with an PSIC score of 2.00 or more, and 58 nsSNPs were predicted to be ‘possibly damaging’ with an PSIC score of 1.40-1.90. The remaining 89 nsSNPs were predicted to be benign.

Sixty-four of the nsSNPs which were predicted to be damaging by SIFT, were also predicted damaging by PolyPhen. rs200639888, rs369532435, rs267602482, rs201234943, and rs377750885 were among the nsSNPs predicted to be highly damaging by SIFT with a tolerance index score of 0.00. These five nsSNPs also have high PSIC scores predicted by PolyPhen (2.439, 2.746, 2.23, 2.373, and 2.46 respectively).

**Fig. 2:**
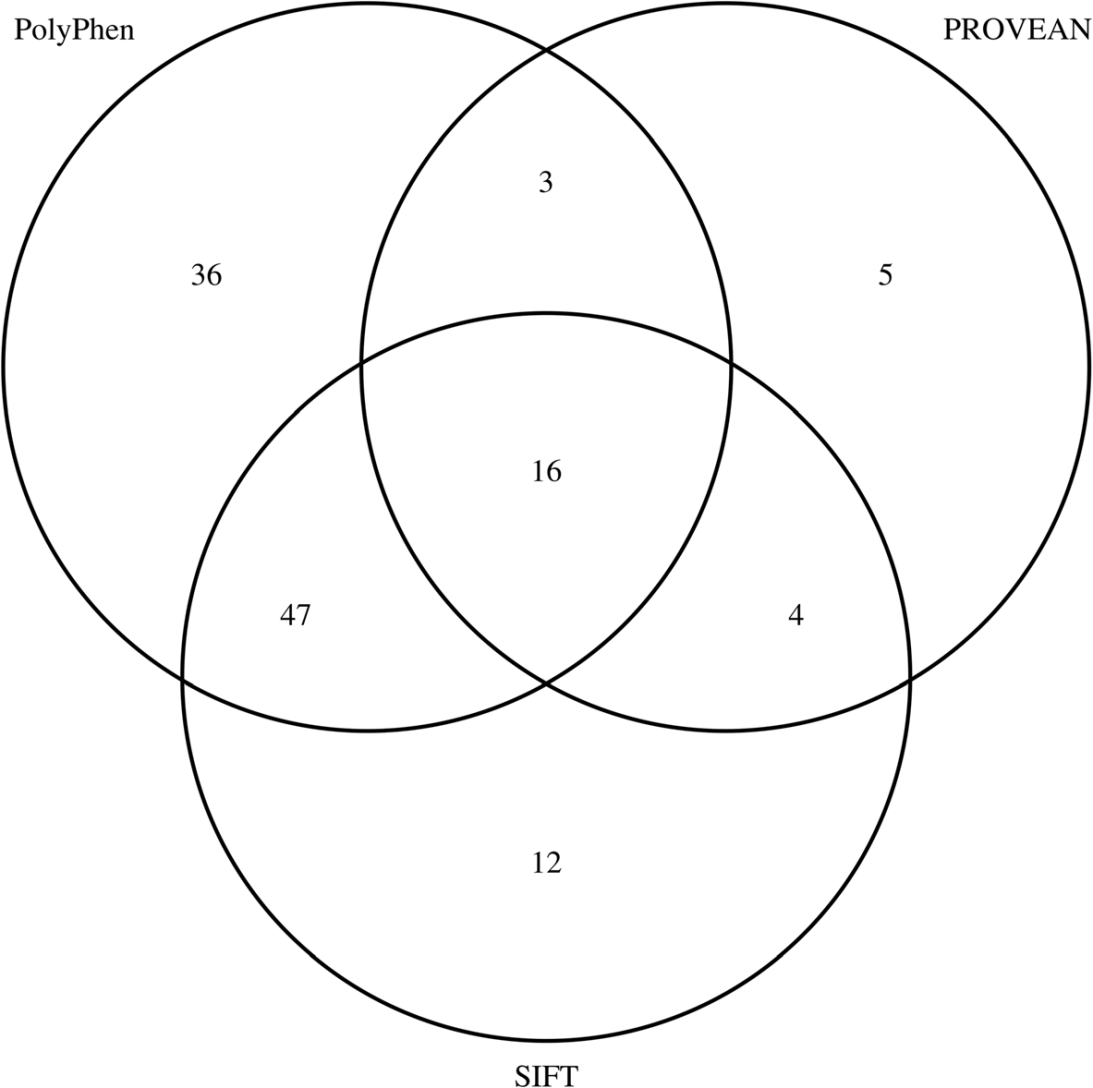
Venn diagram showing the overlap of the predictions made by the three tools PolyPhen, PROVEAN and SIFT

19 nsSNPs were predicted to be damaging by Provean and PolyPhen, and 16 nsSNPs were predicted to be damaging by all three of the used tools (Fig. 2). The nsSNPs rs200639888, rs367841401, and rs377750885 were predicted to be highly damaging/deleterious by all three tools.

### Damaging nsSNPs predicted by at least two tools

As summarized in Table 1, 16 nsSNPs were predicted damaging/deleterious by all three tools, and a total of 69 nsSNPs were predicted damaging by at least two of the used tools. We selected these 69 nsSNPs to perform a more detailed analysis.

**Table 1:** Summary of all 69 nsSNPs predicted 504 to be damaging/deleterious by at least two of the used tools

SNP ID	Location on chromosome	Location in protein	Nucleotide variation	Protein variation	SIFT prediction	Provean prediction	Polyphen prediction	AA change	In ANK domain	Minor Allele Frequency
rs113525905	37447451	613	G/A	G/R	Damaging(0.013)	Neutral(−1.25)	probably damaging(0.998)	hydrophobe > hydrophile		0,19
rs1200875	37505192	985	C/T	R/C	Damaging(0.001)	Neutral(0.32)	possibly damaging(0.762)	hydrophile > hydrophile		12,89
**rs140013037**	37505242	1001	G/C	K/N	Damaging(0.016)	Deleterious(−3.58)	probably damaging(0.963)	hydrophile > hydrophile		0,17
rs144539033	37430978	385	T/C	W/R	Damaging(0)	Neutral(−1.04)	possibly damaging(0.943)	hydrophobe > hydrophile		0,15
rs17590850	37470375	730	A/C	N/H	Damaging(0.002)	Neutral(−0.35)	possibly damaging(0.94)	hydrophile > hydrophile		NA
rs17606645	37470263	723	A/T	K/N	Damaging(0.004)	Neutral(−0.44)	possibly damaging(0.851)	hydrophile > hydrophile		NA
rs183760470	37451752	660	A/C	K/Q	Damaging(0.002)	Neutral(−0.78)	possibly damaging(0.851)	hydrophile > hydrophile		0,01
rs184702413	37481992	838	A/G	E/G	Damaging(0.017)	Neutral(−1.16)	possibly damaging(0.851)	hydrophile > hydrophobe		0,41
rs185294248	37508038	1133	T/C	F/S	Damaging(0.012)	Deleterious(−4.37)	benign(0.006)	hydrophobe > hydrophile		0,11
**rs190686350**	37419160	122	G/A	A/T	Damaging(0.012)	Deleterious(−3.27)	probably damaging(0.997)	hydrophobe > hydrophile	Yes	0,01
rs199571878	37438753	541	A/C	K/Q	Damaging(0)	Neutral(−0.47)	possibly damaging(0.947)	hydrophile > hydrophile		0,079
rs199691521	37488715	926	A/T	E/V	Damaging(0.02)	Neutral(−1.16)	probably damaging(0.994)	hydrophile > hydrophobe		NA
rs199795040	37508139	1167	C/A	Q/K	Damaging(0.002)	Neutral(−2.3)	possibly damaging(0.886)	hydrophile > hydrophile		NA
rs199841724	37508538	1300	C/A	H/N	Damaging(0.032)	Deleterious(−3.11)	benign(0.03)	hydrophile > hydrophile		NA
rs199874591	37451705	644	C/A	P/H	Damaging(0.001)	Neutral(−1.11)	probably damaging(0.997)	hydrophobe > hydrophile		NA
rs200114350	37486388	899	A/G	N/S	Damaging(0.006)	Neutral(−1.64)	possibly damaging(0.713)	hydrophile > hydrophile		NA
rs200264724	37431045	407	C/T	T/M	Damaging(0.001)	Neutral(0.55)	probably damaging(0.989)	hydrophile > hydrophobe		NA
rs200331751	37478422	817	G/A	D/N	Damaging(0.028)	Neutral(−0.12)	possibly damaging(0.818)	hydrophile > hydrophile		NA
rs200399695	37506718	1060	G/C	R/T	Damaging(0.029)	Deleterious(−2.91)	benign(0.013)	hydrophile > hydrophile		NA
**rs200639888**	37419170	125	T/C	L/P	Damaging(0)	Deleterious(−5.96)	probably damaging(0.997)	hydrophobe > hydrophobe	Yes	NA
rs200651327	37418912	105	G/A	E/K	Tolerated(0.081)	Deleterious(−3.37)	probably damaging(0.999)	hydrophile > hydrophile	Yes	NA
rs200845385	37430796	324	C/T	T/I	Damaging(0.002)	Neutral(−0.78)	possibly damaging(0.898)	hydrophile > hydrophobe		NA
**rs200929491**	37508788	1383	G/A	R/H	Damaging(0.002)	Deleterious(−3.55)	probably damaging(0.987)	hydrophile > hydrophile		NA
rs201234943	37447491	626	A/T	K/M	Damaging(0)	Neutral(−1.29)	probably damaging(0.98)	hydrophile > hydrophobe		0,01
rs201628233	37478440	823	G/T	A/S	Damaging(0.022)	Neutral(−0.44)	possibly damaging(0.841)	hydrophobe > hydrophile		0,39
rs201669885	37447325	602	C/G	P/A	Damaging(0.012)	Neutral(−1.66)	possibly damaging(0.924)	hydrophobe > hydrophobe		0,01
**rs201764363**	37508814	1392	G/C	A/P	Damaging(0.002)	Deleterious(−4.03)	probably damaging(0.969)	hydrophobe > hydrophobe		0,01
rs201858051	37508539	1300	A/G	H/R	Tolerated(0.108)	Deleterious(−3.06)	possibly damaging(0.651)	hydrophile > hydrophile		NA
rs201885728	37451744	657	T/C	L/S	Damaging(0.01)	Neutral(−0.08)	possibly damaging(0.932)	hydrophobe > hydrophile		0,01
**rs201943652**	37421175	173	T/C	L/P	Damaging(0.011)	Deleterious(−4.76)	probably damaging(0.995)	hydrophobe > hydrophobe	Yes	NA
rs201976592	37447446	611	C/A	T/N	Damaging(0.002)	Neutral(−1.14)	possibly damaging(0.851)	hydrophile > hydrophile		0,05
rs202090351	37430699	292	C/A	P/T	Damaging(0.001)	Neutral(−1.91)	probably damaging(0.998)	hydrophobe > hydrophile		NA
rs202098264	37430875	350	C/G	F/L	Damaging(0.003)	Neutral(−1.01)	probably damaging(0.965)	hydrophobe > hydrophobe		NA
rs202200263	37454055	679	A/G	D/G	Damaging(0.001)	Neutral(−1.04)	possibly damaging(0.924)	hydrophile > hydrophobe		0,01
**rs267602477**	37419220	142	G/A	A/T	Damaging(0.016)	Deleterious(−3.41)	probably damaging(0.997)	hydrophobe > hydrophile	Yes	NA
rs267602481	37438727	532	C/T	S/F	Damaging(0.001)	Neutral(−1.27)	possibly damaging(0.842)	hydrophile > hydrophobe		NA
rs267602482	37441009	556	C/T	S/F	Damaging(0)	Neutral(−1.66)	probably damaging(0.99)	hydrophile > hydrophobe		NA
**rs267602485**	37507968	1110	G/A	E/K	Damaging(0.021)	Deleterious(−3.34)	probably damaging(0.98)	hydrophile > hydrophile		NA
**rs367841401**	37508002	1121	T/C	L/P	Damaging(0)	Deleterious(−4.47)	probably damaging(0.969)	hydrophobe > hydrophobe		NA
rs368559588	37508121	1161	G/A	A/T	Damaging(0.04)	Neutral(−0.75)	possibly damaging(0.618)	hydrophobe > hydrophile		0,05
rs368660392	37442552	587	A/G	H/R	Damaging(0.003)	Neutral(−1.4)	possibly damaging(0.932)	hydrophile > hydrophile		0,01
**rs369099906**	37508651	1337	A/T	L/F	Damaging(0.001)	Deleterious(−3.22)	probably damaging(0.999)	hydrophobe > hydrophobe		NA
rs369118323	37422851	209	C/T	L/F	Damaging(0.01)	Neutral(−1.52)	probably damaging(0.993)	hydrophobe > hydrophobe	Yes	NA
rs369532435	37438591	519	A/T	K/M	Damaging(0)	Neutral(−1.13)	probably damaging(0.996)	hydrophile > hydrophobe		0,01
rs371253665	37451583	636	C/T	P/L	Damaging(0.001)	Neutral(−0.77)	probably damaging(0.994)	hydrophobe > hydrophobe		0,01
rs371384886	37430859	345	C/T	A/V	Damaging(0.001)	Neutral(−0.56)	probably damaging(0.997)	hydrophobe > hydrophobe		0,01
rs371443557	37431010	395	T/G	I/M	Damaging(0.004)	Neutral(−0.28)	possibly damaging(0.676)	hydrophobe > hydrophobe		NA
**rs371878855**	37508548	1303	A/G	Q/R	Damaging(0.012)	Deleterious(−2.52)	possibly damaging(0.808)	hydrophile > hydrophile		NA
**rs371981371**	37508671	1344	C/A	A/D	Damaging(0.003)	Deleterious(−4.6)	probably damaging(0.989)	hydrophobe > hydrophile		NA
rs372199195	37430803	326	T/G	D/E	Damaging(0)	Neutral(−0.13)	possibly damaging(0.643)	hydrophile > hydrophile		NA
rs372420008	37430922	366	A/G	K/R	Damaging(0.007)	Neutral(−0.62)	possibly damaging(0.956)	hydrophile > hydrophile		NA
rs372878721	37442530	580	G/A	V/M	Damaging(0.013)	Neutral(−0.77)	probably damaging(0.976)	hydrophobe > hydrophobe		NA
**rs373377344**	37508379	1247	G/A	E/K	Damaging(0.048)	Deleterious(−2.78)	possibly damaging(0.898)	hydrophile > hydrophile		NA
rs373380909	37422972	249	G/T	G/V	Damaging(0.003)	Neutral(−2.41)	probably damaging(0.999)	hydrophobe > hydrophobe	Yes	NA
**rs373997768**	37505217	993	A/C	K/T	Damaging(0.003)	Deleterious(−2.76)	probably damaging(0.963)	hydrophile > hydrophile		NA
rs374024060	37430943	373	C/T	T/M	Damaging(0.011)	Neutral(−0.76)	probably damaging(0.975)	hydrophile > hydrophobe		NA
rs374037740	37441038	566	T/G	W/G	Damaging(0.009)	Neutral(−1.82)	possibly damaging(0.826)	hydrophobe > hydrophobe		NA
rs374739457	37454063	682	G/C	E/Q	Damaging(0.018)	Neutral(−0.76)	possibly damaging(0.851)	hydrophile > hydrophile		NA
rs374753521	37508446	1269	A/C	Y/S	Damaging(0.031)	Deleterious(−4.18)	benign(0.347)	hydrophile > hydrophile		NA
rs375945698	37505306	1023	G/C	E/Q	Damaging(0.018)	Neutral(−2.17)	probably damaging(0.999)	hydrophile > hydrophile		NA
rs376116213	37505157	973	G/A	R/K	Damaging(0.004)	Neutral(−2.23)	probably damaging(0.976)	hydrophile > hydrophile		0,01
rs376821949	37438772	547	G/A	R/K	Damaging(0)	Neutral(0.04)	possibly damaging(0.643)	hydrophile > hydrophile		NA
rs377410013	37440994	551	T/C	M/T	Damaging(0.045)	Neutral(−0.42)	possibly damaging(0.717)	hydrophobe > hydrophile		NA
rs377740138	37430720	299	G/A	V/M	Damaging(0.002)	Neutral(−0.39)	possibly damaging(0.845)	hydrophobe > hydrophobe		NA
rs377744149	37508352	1238	G/A	D/N	Tolerated(0.083)	Deleterious(−3.19)	probably damaging(0.971)	hydrophile > hydrophile		0,01
**rs377750885**	37508803	1388	A/T	E/V	Damaging(0)	Deleterious(−5.87)	probably damaging(0.997)	hydrophile > hydrophobe		NA
rs41276130	37451768	665	T/G	L/W	Damaging(0.002)	Neutral(−0.98)	probably damaging(0.983)	hydrophobe > hydrophobe		4,17
rs45515098	37440991	550	C/T	P/L	Damaging(0.028)	Neutral(−1.27)	possibly damaging(0.581)	hydrophobe > hydrophobe		0,01
**rs61737412**	37419218	141	C/T	T/M	Damaging(0.035)	Deleterious(−5.03)	possibly damaging(0.951)	hydrophile > hydrophobe	Yes	4,13

The 16 nsSNPs in bold letters were predicted damaging/deleterious by all three used tools

**Fig. 3:**
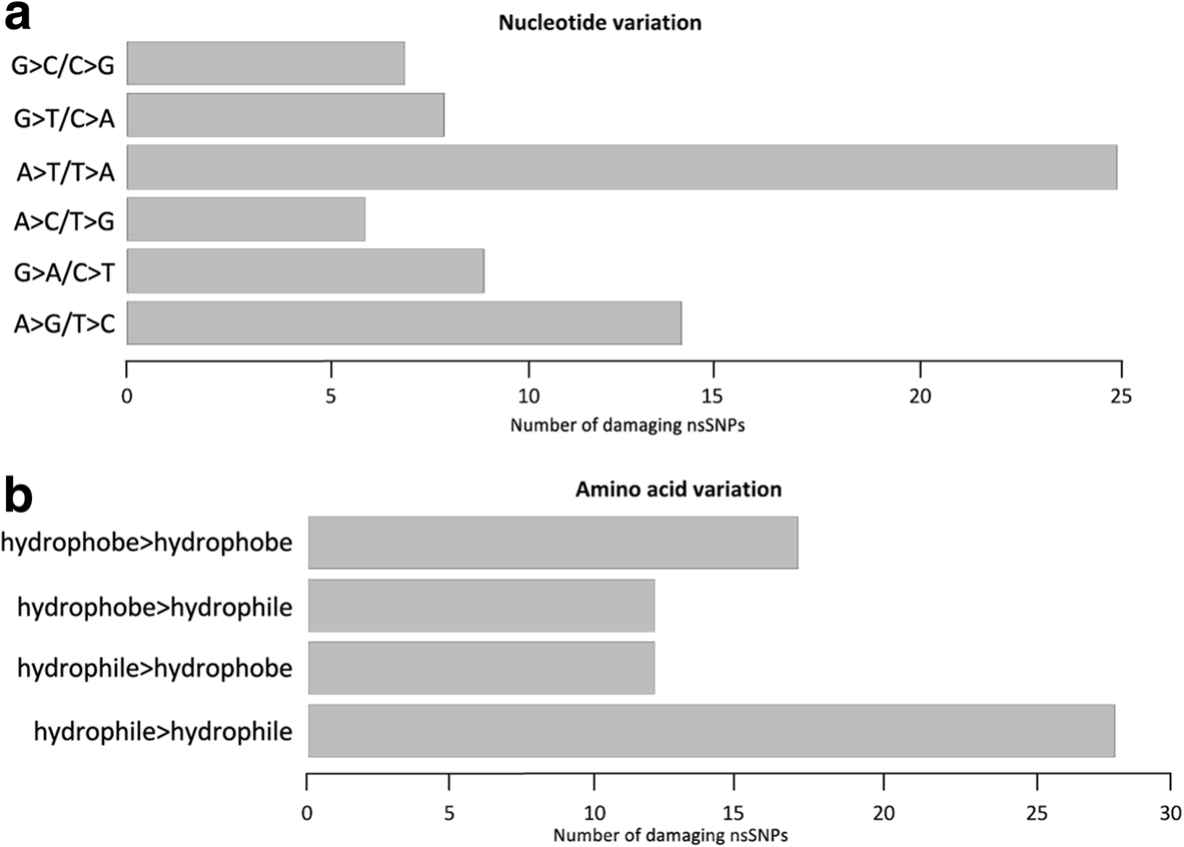
Graphical representation of spectrum of damaging nsSNPs variation. **a**) nucleotide variations, **b**) amino acid variations

Analysis of the spectrum of nsSNPs on the nucleotide level showed a conserved profile with A > T/T > A transitions and hydrophile > hydrophile transitions being the most frequent changes (Fig. 3a and b).

The nsSNPs rs200639888 and rs367841401, which were predicted to be highly damaging by all three tools, have an amino acid change from leucine to proline which are both hydrophobe amino acids. The third damaging nsSNP predicted by all three tools, rs377750885, has a change from glutamic acid (hydrophile) to (hydrophobe) valine.

The minor allele frequency describes the proportion of the least common allele in a certain population pool. Table 1 summarizes the minor allele frequency for the 69 nsSNPs, predicted to be deleterious by two tools. Allele frequencies are only provided for 26 of the analyzed SNPs. For most SNPs the minor allele frequency is below 1% except for the SNPs rs1200875 (12.9%), rs41276130 (4.17%), and rs61737412 (4.13%).

### Clinical data analysis

As part of an in-house cancer sequencing project, exome-sequencing data was available for 55 breast cancer patients and was provided for analysis in this study. In the analyzed patient cohort 11 SNPs were detected in in the NY-BR-1 gene: rs34042320, rs1209750, rs34552277, rs61737412, rs41276130, rs1200876, rs1200875, rs41304589, rs116939015, and rs16937417 (Table 2). Seven SNPs occur in more than 10 patients and three of these (rs61737412, rs41276130, rs1200875) were predicted damaging by at least two of the used tools. These SNPs also have a high minor allele frequency of 4.13, 4.17, and 12.89, respectively. The SNP rs1209750 occurs in 49 patients, which corresponds to almost 90% of analyzed patients. rs1209750 also has a high minor allele frequency of 48,22%. This SNP however, was not predicted to be damaging. Likewise, the SNPs rs1200876, rs34042320, and rs34552277 occur in a large fraction of the patient cohort and also have a high minor allele frequency. These SNPs were also not predicted to be damaging. A Fisher’s exact test was performed to test the difference in allele frequencies of the SNPs in our breast cancer patient cohort against the dbSNP reference for significance which showed six SNPs to be significantly enriched in the analyzed patient cohort.

**Table 2:** SNPs and mutations detected in the analyzed breast cancer patient cohort of 55 patients

SNP ID	Prediction	Frequency in Patient Cohort	Minor Allele Frequency in dbSNP	p-value (Fisher’s exact test)
rs116939015		1,82	0,59	0,4921
rs1200875	damaging	45,45	12,89	0,0018
rs1200876		45,45	12,77	0,0016
rs1209750		89,09	48,22	0,0149
rs12766884		1,82	4,432	0,0853
rs16937417		1,82	2,61	0,3646
rs34042320		18,18	3,27	0,0033
rs34552277		38,18	22,76	0,3381
rs41276130	damaging	21,82	4,17	0,0020
rs41304589		9,09	2,55	0,2059
rs61737412	damaging	27,27	4,13	3,80E-05
somatic (chr10:37430943:C/T)		1,82	NA	
somatic (chr10:37447328:A/G)		1,82	NA	

Two somatic mutations were also detected in the patient cohort which both occur only in single patients. The somatic mutation chr10:37430943:C > T translates to a T > M transition at position 317 in the NY-BR-1 protein. This mutation is also documented in the Catalogue of Somatic Mutations in Cancer (COSMIC) [22] database as COSM4137978 and was reported in two patients with ovary cancer. The second somatic mutation chr10:37447328:A > G translates to a N > D transition at protein position 547 and is not documented in the COSMIC database.

## Discussion

Information on genetic variation can provide a valuable insight into the functional range and critical regions of a gene. SNPs are the most common form of genetic variations and a link between SNPs and complex diseases have been reported for a number of cases. The BRCA-1 gene for example and some of its interaction partners are associated with breast cancer. SNPs in these genes are not just involved in the onset of a disease but they can promote also disease progression and outcome [23, 24]. Here, we systematically analyzed SNPs in the NY-BR-1 gene to identify those SNPs which can modify the functional properties of the protein.

In the Ensembl BioMart database 2898 SNPs are recorded for the NY-BR-1 transcript ENST00000611781. Out of these, 191 (6.01%) were nonsynonymous SNPs (nsSNPs), i.e. polymorphisms which translate into an altered amino acid sequence. As these types of SNPs are most likely to have an effect on protein function, we chose to analyze only them further.

Computational approaches use various types of features to predict the functional impact of nsSNPs: physical and chemical properties of the affected amino acids, structural properties of the encoded protein, and evolutionary properties, which can be inferred from sequence alignments of homologous proteins. We chose three state-of-the-art computational tools which can predict the effects of amino acid substitutions on protein function: SIFT, Provean and PolyPhen2.

**Fig. 4:**
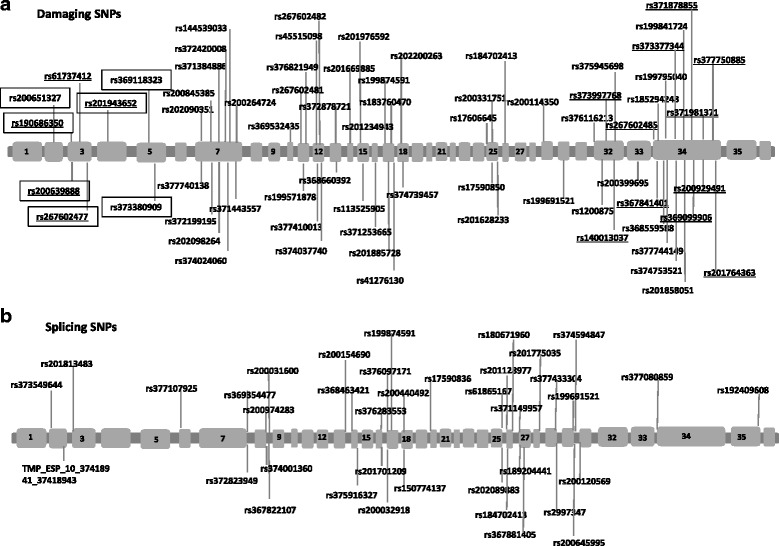
Graphical representation of location of NY-BR-1 SNPs. **a**) damaging SNPs, **b**) splicing SNP. SNPs predicted damaging by all three tools are underlined and SNPs located in an ANK repeat domain are highlighted with a box

191 nsSNPs were analyzed and the results varied between the used tools: SIFT predicted 79 damaging nsSNPs, Provean 28 nsSNPs, and PolyPhen2 102 nsSNPs. 16 nsSNPs were predicted damaging by all three tools, and a total of 69 nsSNPs were predicted damaging by at least two of the used tools. SIFT and PolyPhen2 have the biggest overlap with 63 common predictions. This may be due to the common step of assessing the degree of conversation by utilizing a multiple sequence alignment of homologous sequences. 36 damaging nsSNPs were only predicted by PolyPhen2 because PolyPhen2 is the only tool that takes functional relevant sites into account. The location of the 69 damaging SNPS within the ANKRD30A gene is shown in Fig. 4a.

Up to date the structure of NY-BR-1 has not been solved yet and no homology models for the entire protein are available. Thus, we unfortunately could not evaluate the location and effect of the predicted damaging nsSNPs on the protein structure.

In the UniProt database six ankyrin (ANK) repeat motifs are documented for NY-BR-1. The ANK repeat motif is one of the most common protein-protein interaction motifs in nature and occurs in a large number of functionally diverse proteins. The structure of the ANK repeat motif is conserved: each repeat typically consists of 30–34 amino acid residues comprising two anti-parallel α-helices and a long loop ending in a β-hairpin [25]. Proteins containing the ANK repeat motif are involved in a diverse set of cellular functions, and defects in ANK repeat proteins have been associated with a number of human diseases [26, 27]. Hence, a variation within such a functional domain is likely to have an impact on protein function.

Eight of the 69 damaging nsSNPs in NY-BR-1 are located in an ANK repeat motif: rs190686350, rs200639888, rs61737412, rs267602477, rs201943652, rs200651327, rs369118323, rs373380909. PolyPhen2 predicted all of them as damaging, whereas Provean predicted two, and SIFT one of them as not damaging. SNPs influencing the splicing process also may have an impact on protein function if the newly generated transcripts are translated into proteins. In dbSNP, 39 of the NY-BR-1 SNPs are annotated to be splicing, located at donor or acceptor sites (Fig. 4b). These SNPs have the potential to influence the splicing process and thus give rise to new transcripts.

An unknown fraction of SNPs submitted to the public databases may not be true polymorphisms, but examples of sequencing errors. Therefore it is important to consider the validation status of each SNP. A polymorphism can be validated by independent submissions or frequency/genotype data. In our dataset 1832 out of 2898 SNPs have been validated. Considering the 69 damaging nsSNPs, 16 have not been validated yet. As these nsSNPs seem to have an impact on protein function, validation of them should especially be considered.

Allele frequencies are only provided for 26 of the analyzed SNPs 69 nsSNPs, predicted to be damaging by at least two tools. SNPs with no information on allele frequencies are usually based on single submissions, often from sequencing projects of cancer patient cohorts and therefore might be of special relevance. The minor allele frequency of 16 out of 26 analyzed SNPs is below 0.1%, for six analyzed SNPs the minor allele frequencies are between 0.1% and 1%, and for three SNPs the minor allele frequency is greater than 4% According to Frazer et al. these SNPs can be classified according to their minor allele frequencies: variants with minor allele frequencies between 0.1% and 3% were defined as rare variants, variants with minor allele frequencies of less than 0.1% as novel variants, and high-frequency common variants were defined as variants with minor allele frequencies greater than 5% [28].

We also analyzed in house exome-sequencing data of 55 breast cancer patients and as expected, NYBR1 was found to be expressed in all patients (data not shown). Somatic mutations were only detected in two patients. Also, as indicated by the database research on COSMIC, the two somatic mutations are not being frequently observed in cancer patients. Only one of the mutations, COSM4137978, is documented in COSMIC and was observed in two cases of ovary cancer. Also using the COSMIC database, we further searched for NY-BR-1 mutations in breast cancer patients. Only 27 out of 1436 breast cancer patients were found to have a somatic mutation in NY-BR-1 indicating that somatic mutations in this gene is not a frequent event in breast cancer patients.

In contrast, 11 SNPs in the NY-BR-1 gene were found in the in-house patient cohort, seven SNPs occurring in more than 10 patients. Three of these frequent SNPs (rs61737412, rs41276130, rs1200875) were also predicted damaging by at least two of the used tools. These SNPs also have a high minor allele frequency in dbSNP, they are however highly enriched in the patient cohort (p-value < 0.002, Fisher’s exact test). There are also three other SNPs (rs1200876, rs1209750, rs34042320), that are enriched in the patient cohort (p-value < 0.01, Fisher’s exact test), but these SNPs were not predicted damaging. These SNPs which seem to be enriched in breast cancer patients need to be further analyzed in larger patient cohorts to elucidate whether there is a correlation to clinical status and outcome. The effect of these SNPs on protein function also still needs to be determined.

## Conclusion

In summary, we have identified 69 damaging nsSNPs within the coding region of the breast cancer associated NY-BR-1 gene. Moreover, we found 39 potential splicing SNPs which can affect the alternative splicing process. Our analysis gives an overview on the SNP landscape of NYBR1 and now provides the basis to further study the association of SNPs and the molecular breast cancer subtypes “Her2”, “Luminal A/B” and “Triple negative” as well as clinical data, such as treatment response, relapse rate and overall survival.

## Abbreviations

ANK: Ankyrin
indels: Small insertions and deletions
NY-BR-1: New York-Breast-1
SEREX: Serological cloning strategy
SNP: Single Nucleotide Polymorphism
